# A Discussion on Perineal Tears

**Published:** 1907-09

**Authors:** John Egerton Cannaday

**Affiliations:** Surgeon-in-Charge, Sheltering Arms Hospital, Hansford, W. Va.


### A Discussion on Perineal Tears.

BY JOHN EGERTON CANNADAY, M.D.
Surgeon-in-Charge, Sheltering Arms Hospital, Hansford, W. Va.

(*American Journal of Obstetrics—Author's Abstract.*)

THE author reviews the history of the subject and says, "The literature is enormous if not appalling. Innumerable operations have been proposed and practised and almost every suture devised by man has been tried; all eloquent to the fact that none of the methods are perfect. A perusal of some of the writings on the subject would tend to confuse the mind of the reader with their intricacies, but when shorn of its complexities and reduced to the basic principles of surgery a perineal tear revolves itself into a comparatively simple matter."

The anatomy of the parts concerned is taken up in detail, the author believing that a thorough understanding of this part of the subject makes the matter a comparatively simple one to the operator. In speaking of the supports of the pelvic organs the author says, "A number of widely differing views as to what factors normally enter into the support of the pelvic contents are held by the principal authorities of the world. Some hold that the levator ani with or without the aid of the other muscles is the chief power for support. Others ascribe all virtue to the fascia. Some, again, give both muscles and fascia more or less equal credit for accomplishing the work of support between them. Personally the author believes that the muscles with the fascia act as a composite diaphragm in closing the lower end of the abdominal cavity and in giving support to the pelvic organs. Of the two factors he considers the fascia of the major importance; in the anterior abdominal wall we consider the fascia the utmost importance in the prevention of hernia. The pelvis should not be radically different." The indications for repair are given and the operation is minutely described. The author shows that the denudation outline is practically that of the capital letter M, the outline of sutures representing the letter Y. The various methods of different operators are described.

The advantages of feeding the patient on minimum amounts of albumin and of locking the bowels for two weeks in the after treatment of complete tear cases are stated.

